# Kinomic profiling to predict sunitinib response of patients with metastasized clear cell Renal Cell Carcinoma

**DOI:** 10.1016/j.neo.2024.101108

**Published:** 2024-12-25

**Authors:** Jeannette C. Oosterwijk-Wakka, Liesbeth Houkes, Loes F.M. van der Zanden, Lambertus A.L.M. Kiemeney, Kerstin Junker, Anne Y Warren, Tim Eisen, Ulrich Jaehde, Marius T Radu, Rob Ruijtenbeek, Egbert Oosterwijk

**Affiliations:** a Radboud University Medical Center, 6525 GA, Nijmegen, the Netherlands; b PamGene International B.V., 5211 HH 's-Hertogenbosch, the Netherlands; c Clinic of Urology and Paediatric Urology, Saarland University, 66424 Homburg, Germany; d Department of Oncology, Cambridge University Hospitals NHS Foundation Trust and Cambridge Biomedical Research Centre, Cambridge CB2 0QQ, UK; e Department of Histopathology, Cambridge University Hospitals NHS Foundation Trust and Cambridge Biomedical Campus, Cambridge CB2 0QQ, UK; f CESAR Central Office, CESAR Central European Society for Anticancer Drug Research-EWIV, 1010, Vienna, Austria; g University of Medicine and Pharmacy Carol Davila 050474, Bucharest, Romania

**Keywords:** Renal Cell Carcinoma, TKI, biomarker, kinase activity

## Abstract

**Introduction:**

Treatment with Sunitinib, a potent multitargeted receptor tyrosine kinase inhibitor (TKI) has increased the progression-free survival (PFS) and overall-survival (OS) of patients with metastasized renal cell carcinoma (mRCC). With modest OS improvement and variable response and toxicity predictive and/or prognostic biomarkers are needed to personalize patient management: Prediction of individual TKI therapy response and resistance will increase successful treatment outcome while reducing unnecessary drug use and expense. The aim of this study was to investigate whether kinase activity analysis can predict sunitinib response and/or toxicity using tissue samples obtained from primary clear cell RCC (ccRCC) from a cohort of clinically annotated patients with mRCC receiving sunitinib as first-line treatment.

**Materials and Methods:**

EuroTARGET partners collected ccRCC and matched normal kidney tissue samples immediately after surgery, snap-frozen and stored at -80°C until use. Phosphotyrosine-activity profiling was performed using PamChip® peptide microarrays (144 peptides derived from known phosphorylation sites in Protein Tyrosine Kinase substrates) of lysed tissue samples (5 µg protein input) of 163 mRCC patients. Evolve software Was used to analyze kinome profiles and Bionavigator was used for unsupervised and supervised clustering. The kinexus kinase predictor (www.phosphonet.ca) was used to analyze the peptide lists within the clusters.

**Results:**

Kinome data was available from 94 patients who received sunitinib as 1st-line treatment and had complete follow-up of their clinical data (PFS, OS and toxicity) for at least 6 months. Matched normal tissue was available from 14 mRCC patients. Supervised clustering of basal kinome activity could correctly classify mRCC patients with PFS >9 months *versus* PFS<9 months with an accuracy of 61 %. Unsupervised hierarchical clustering revealed 3 major clusters related to immune signaling, VEGF pathway, and immune signaling/cell adhesion. Basal kinase activity levels of patients with short PFS were substantially higher compared to patients who experienced extended PFS.

**Discussion/Conclusion:**

Based on kinase levels ccRCC tumors can be subdivided into 3 clusters which may reflect the aggressiveness of these tumors. The accuracy of response prediction of 61 % based on basal kinase levels is too low to justify implementation. STK assays may help to predict sunitinib toxicity and guide clinical management. Additionally, it is possible that mRCC patients with an immune kinase signature are better checkpoint inhibitor candidates, but this needs to be studied.

## Introduction

Renal Cell Carcinoma (RCC) accounts for 2-3 % of all malignancies [1]. Approximately 50 % of all patients have metastasized renal cell cancer (mRCC) at presentation or develop metastases during follow-up. Survival of mRCC patients has been extremely poor (5-10 % 5-year survival), but treatment with tyrosine kinase inhibitors (TKI) that suppress angiogenesis and mammalian target of rapamycin (mTOR) inhibitors, have increased the progression free survival (PFS) and overall survival (OS) [2,3]. Recently, immune-checkpoint inhibitors (IC) have been added to the treatment regimen of patients with mRCC superseding TKI as 1st line treatment [4]. However, in patients who cannot tolerate this treatment, sunitinib is one of the first line treatment modalities for all mRCC risk groups [5]. Response and toxicity to sunitinib and other expensive drugs is extremely variable. To personalize patient management predictive and/or prognostic biomarkers are needed: this will increase successful treatment outcome while reducing unnecessary drug use and expense. Minimally invasive prognostic biomarkers such as elevated neutrophil-to-eosinophil ratio and the Royal Marsden Hospital score (based on blood variables and clinical features) may be of value: they are associated with poorer OS and PFS in cancer patients (including RCC) as shown in systematic review and meta-analysis [6,7]. However, such markers are general in nature and therefore it is unlikely that they can serve as biomarker for a drug specific response.

Several studies examined whether molecular mechanisms were associated to sunitinib resistance and toxicity profiles in mRCC: whole exome sequencing of extreme phenotypes suggested a potential association between somatic *PBRM1* mutations and favorable response [8]; sunitinib failure has been related to aberrant miRNA expression [9]; *AXL* and c*-MET* expression through lncRNA regulating miR-34/miR-449 might confer sunitinib resistance [10]; restoration of TK-independent alternate angiogenesis pathways might lead to sunitinib resistance [11], and Hedgehog-related signaling has been implicated as a predictor of disease progression following treatment with sunitinib [12] (reviewed in [13]). Because the mechanism of action of TKI is at the level of protein phosphorylation, signal transduction and thus kinase activity, kinase activity profiling of clear cell RCC (ccRCC) may permit a better prediction of TKI sensitivity and/or toxicity than molecular events. Kinase profiling of primary RCC from patients undergoing surgery for localized disease already revealed three distinct kinome clusters [14]. How these relate to response to TKI therapy is at present unresolved. Additionally, kinase analysis showed that both sunitinib and the active metabolite SU12662 induced TK inhibition in peripheral blood mononuclear cells (PBMC) [15]. The study suggested that a stronger ex vivo inhibition of the PBMC kinome profile before sunitinib start might be associated with a better prognosis in mRCC patients.

In the current study we focused on tissue-based predictive markers, i.e., investigated whether kinase activity analysis could predict sunitinib response and/or toxicity, using tissue specimens obtained from primary ccRCC from a large cohort of clinically annotated patients with mRCC receiving sunitinib as first line treatment modality collected by the EuroTARGET consortium [16].

## Material and Methods

Fresh-frozen ccRCC and matched normal kidney tissue (when available) were provided by EuroTARGET partners. EuroTARGET was an EU 7th framework program project aiming to identify and characterize host and tumor related biomarkers for prediction of response to TKI therapy in mRCC patients. The characteristics of the complete EuroTARGET cohort (1210 mRCC patients, 920 receiving TKI as first line treatment) have been described elsewhere [16]. Registration of detailed clinical information from all patients (baseline and follow-up) was entered in web-based case record forms. Tissue for kinase analysis was available from 163 patients. Surgical specimens were snap-frozen and stored at the local biobanks until use. Central pathology review was performed for all samples. All tumors analyzed were ccRCC. For the current analysis, only specimens containing >80 % tumor cells and <35 % necrosis as judged by HE, were included. Sections of 20 µm were sent to the central biobank repository at Radboudumc, The Netherlands and stored at −80°C until use. Minimal follow-up was 6 months after initiation of TKI treatment.

To exclude putative batch-to-batch differences, 163 fresh-frozen ccRCC samples were profiled simultaneously in 3 technical replicates. Samples from patients who did not receive sunitinib as first line TKI treatment, had follow-up of less than 6 months and samples that contained more than 50 % necrosis were excluded from the analysis. In addition, samples with kinase activity >10-fold lower than the highest median activity or 2.7 times lower than the mean of all activities were excluded from the analysis. Matched normal tissue was available from 14 mRCC patients that experienced toxicity grade 2 or higher.

### Kinase activity profiling

The PamChip assay quantifies kinase activity in cell and tissue lysates by measuring the phosphorylation of peptide representations of kinase targets/ substrates (referred hereafter as phosphosites) that are immobilized on the PamChip® microarrays. The Tyrosine and Serine/Threonine kinase activity profiling was performed using a PamStation®96 platform (PamGene International B.V.,’s Hertogenbosch, The Netherlands) and PTK or STK PamChip®96 with 96 identical arrays (PTK, product 86311, STK, product 87101, PamGene). Samples (10 × 10 µm slides) were homogenized in M-PER mammalian protein extraction reagent containing 1x HALT protease inhibitor cocktail and 1x HALT phosphatase inhibitor cocktail (Fisher Scientific, Landsmeer, The Netherlands) and the protein concentration was determined by the Coomassie Plus method according to the manufacturers’ instructions (Fisher Scientific, Landsmeer, The Netherlands). The incubation mixture was composed of 5µg of protein input, equivalent to approximately 1/20 mm^3^ tissue, 1xPK buffer for protein kinases (New England Biolabs, Ipswich, Massachusetts, USA), 0.01 % BSA (Calbiochem, Darmstadt, Germany), 10mM DTT (Sigma-Aldrich, Houten, The Netherlands), 1x PTK additive (PamGene 20150219NBa), 15mM MgCl (Sigma-Aldrich, Houten, The Netherlands), 100 μM ATP and a FITC-conjugated PY20 (BioRad, Veenendaal, The Netherlands, Batch1114, F/P ratio 2.7) in 3 technical replicates. Where mentioned, sunitinib (0, 0.03, 0.1, 0.3 µM) (Selleckchem, Bio-Connect BV, Huissen, The Netherlands) was spiked into the mixture. The mixture was pumped up and down through the porous ceramic membrane and peptide phosphorylation was monitored real-time with a fluorescence CCD camera in combination with Evolve software version 1.5. (PamGene). The arrays were washed with 1xPK buffer for protein kinases and peptide spot intensity was captured at different exposure times (20, 50, 100 and 200 ms). Images were used by the BioNavigator® software to calculate signal values for each phosphosite. The detection in the STK assay was performed with 3 primary STK antibodies (Cell Signaling technology, #9624, #9614, #2325 Rabbit mAb) and a FITC-conjugated Polyclonal Swine Anti-Rabbit secondary antibody (Dako, Glostrup, Denmark, F0205).

### Analysis

First, the quality of the dataset was assessed based on PamGene's QC criteria for signal strength (>300 AU), number of peptides (>90) and the replicate variation (<20 %). The quality scores serve to assess the reliability of the data. Images were obtained every 5 min during the assay (kinetic readout). Only phosphosites that showed kinetics (increased signal in time) on at least 25  % of the arrays were included in the downstream analysis. Kinomic profiles were analyzed using Evolve software (PamGene) for initial sample and array processing and image analysis. BioNavigator® (PamGene) was used to transform raw data into kinetic (initial velocity) and steady state (postwash) values. By using multiple exposure time integration, the dynamic range of the measurements is increased.

To identify significant differences in the conditions at the phosphosite level, the statistical tests mentioned below were used:

1. Unsupervised hierarchical clustering of Log2 transformed signals.
2. Supervised analyses of patients at progression 6 months post-initiation of sunitinib treatment
3. Partial least squares-discriminant analysis (PLS-DA)

Unsupervised hierarchical clustering of peptide signals was performed in BioNavigator® and was displayed as heatmaps. The visual assessment of overall signal resulted in Log2-transformed values for each peptide (rows) and each array (columns). The heatmap visualization provides an overview of samples and measurements. This view helps to indicate any possible trends and outliers. Each row represents a phosphosite ad each column a patient sample. Rows were sorted by row mean values and only include phosphosites which passed the QC. To correct for signal intensity differences, variance stabilizing normalization (VSN) was implemented. This normalization method allows for the comparison of large numbers of samples especially from multiple PamStation runs or human samples. Therefore, this method is ideally suited for PamChip assays. The consequence of VSN is that the data focuses on the relative differences between samples rather than the absolute differences.

The Upstream Kinase Analysis (UKA) algorithm was used to predict differential kinase activity between groups. UKA, a data analysis pipeline, and part of the BioNavigator software tool developed by PamGene, uses data from publicly available databases that specify kinase-to-substrate relationships. Therefore, the interpretation of the derived kinases from UKA is highly dependent on the contents of these databases. Ultimately, the results from UKA can be used to generate hypotheses. The selected kinases need to be further validated using different approaches. The peptide lists within the clusters were analyzed for upstream kinase prediction using the Kinexus Kinase Predictor (www.phosphonet.ca) and in-house bioinformatics toolbox to generate a list of top altered kinases. Network modeling of clusters was performed by uploading the parent protein Uniprot ID for all peptides higher in their respective cluster to MetaCore software (GeneGo ™, portal.genego.com, Thompson Reuters).

In the supervised analyses, patients that progressed < 9 months post-initiation of sunitinib treatment were compared to non-progressing patients, and patients that showed toxicity grade 2 or higher were compared with patients with toxicity grade 0 or 1. An unpaired student's t-test was used to identify significant peptide-phosphorylation differences (*p* < 0.05). The peptides that distinguished patient groups in the supervised clustering were also analyzed using the upstream kinase predictor strategy as described above. In addition, to analyze sunitinib toxicity, 14 ccRCC tumor specimens were directly compared with matched normal kidney tissue specimens at the kinome level using a student's t-test (7 specimen with low/no toxicity and 7 with toxicity level 3-4). Significantly altered peptides (*p* < 0.05) were used for upstream kinase prediction analysis as described above. Additional analyses of kinomic and clinical data included Fisher's Exact Testing of progression status and kinomic groups.

Kaplan-Meier plots were generated in SPSS (IBM SPSS statistics 27).

## Results

**Fig. 1:**
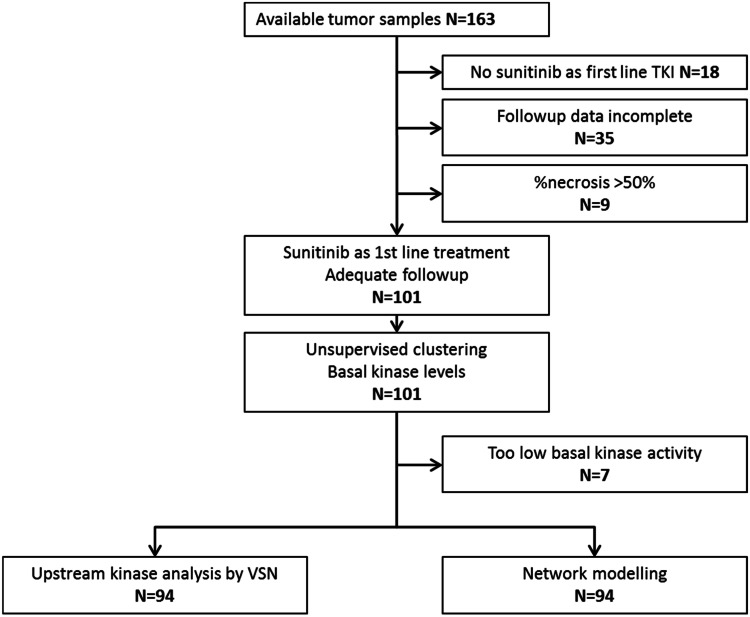
Flow diagram of study design. One hundred **one** fresh-frozen tumor tissue samples of patients with mRCC receiving sunitinib first line were included in the study.

Kinase activity profiling using peptide microarrays (both Tyrosine and Serine/Threonine kinase activity) was performed to investigate whether kinase activity analysis can predict response or toxicity to sunitinib. Primary tumor tissue specimens obtained from 163 patients with mRCC before the patients received sunitinib treatment were collected by the EuroTARGET consortium: 138 males (age 60.8 ± 9.1 year at start of TKI treatment and 25 females (age 62 ± 12.5 year at start of TKI treatment). Eighteen samples were from patients who did not receive sunitinib as first line TKI treatment, 35 samples were from patients for whom follow-up for at least 6 months was absent and 9 samples contained more than 50 % necrosis and were therefore excluded from the analysis. Of the remaining 101 samples, 7 had kinase activity >10-fold lower than the highest median activity or 2.7 times lower than the mean of all activities and were therefore excluded from the analysis. In total, kinome data were available from 94 patients who received sunitinib as first line treatment and had complete follow-up of their clinical data (PFS, OS, toxicity) for at least 6 months. Matched normal tissue was available from 14 mRCC patients that experienced toxicity grade 2 or higher. This cohort of analyzed patients consisted of 13 males (mean age 59.9 ± 9.4 years) and 1 female (age 53). See Fig. 1.

**Fig. 2:**
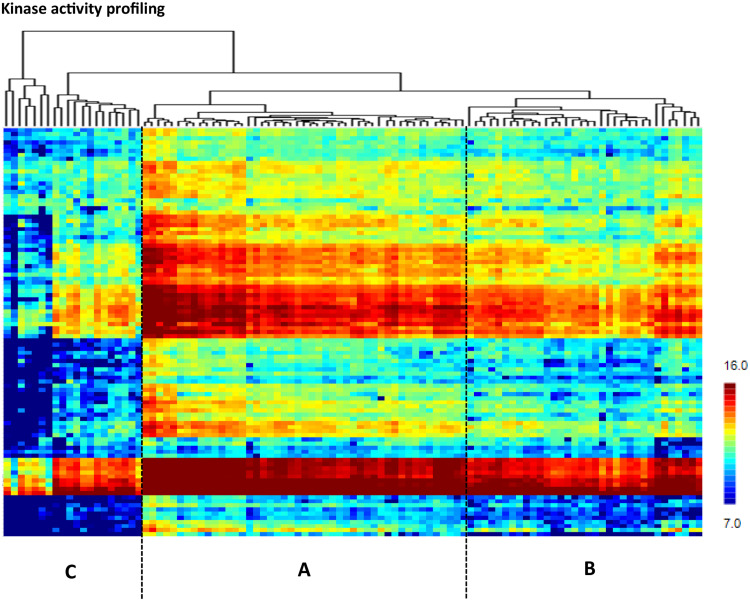
Unsupervised hierarchical clustering of peptide phosphorylation (Y-axes) of 101 primary RCC from metastatic ccRCC patients pre-sunitinib treatment revealing 3 major clusters (A, B and C).

Unsupervised hierarchical clustering of kinomic profiles revealed 3 major clusters (Fig. 2): Cluster A (*N* = 47, 47 %), Cluster B (*N* = 34, 34 %), Cluster C (*N* = 20, 20 %).

Z-score normalized per peptide (suppl. **Figure S1**) showed that 38 peptides were significantly higher phosphorylated in cluster A versus cluster B (suppl. Table S1A), 24 peptides were relatively higher phosphorylated in cluster B versus cluster A (Suppl. Table S1B), 29 peptides were significantly differently higher phosphorylated in cluster B versus cluster C (suppl. Table S1C) and 15 peptides were relatively higher phosphorylated in cluster C versus cluster B (Suppl. table S1D).

Pathway analysis using MetaCore software revealed that kinase activities related to the immune signaling, such as Fyn, ZAP70, Src, Ron and Syk were highly upregulated in cluster A (Suppl table S2A, S3A). Kinase activity related to EGFR, EphA2, Fgr, FLT4, FLT1, FLT3 within the MAPK signaling pathway were more prominent in cluster B compared to A (Suppl table S3B). We observed upregulation of kinase activities related to immune signaling (Fyn, Ron, HCK, BLK and Syk) as well as to cell adhesion Ephrin in cluster B compared to cluster C (Suppl table S2B, S3C), whereas in cluster C kinase activities within the VEGF signaling pathway, such as FLT1,3,4, were upregulated (Suppl Table S3D).

**Fig. 3:**
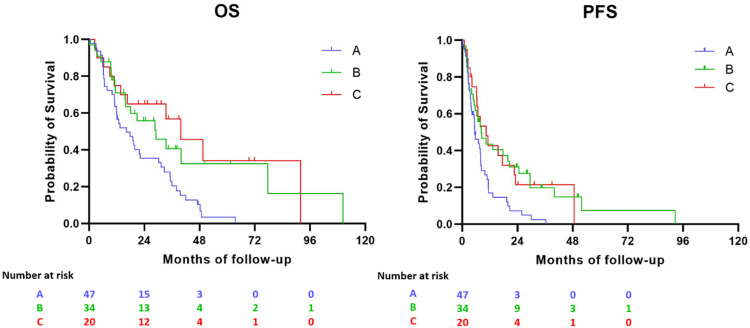
Kaplan–Meier plots and risk tables showing overall survival (A) and PFS (B) of mRCC patients per cluster. Number of patients at risk are shown. Patients in cluster B and C survived significantly longer (*p* < 0.005 and *p* < 0.05 for OS and PFS respectively).

Next, the PFS and OS per cluster group were analyzed (Fig. 3). Patients with a cluster B and cluster C kinome signature outperformed patients with cluster A kinome signature (*p* < 0.02 ANOVA).

## Supervised analyses

**Fig. 4:**
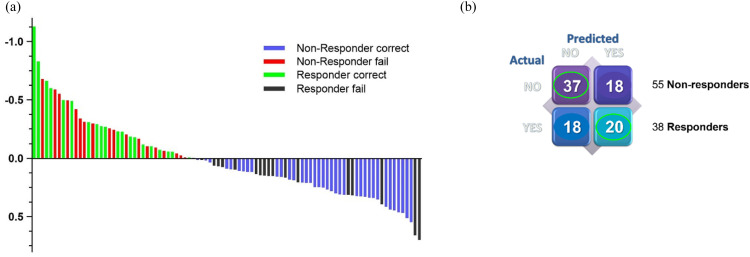
**Supervised prediction of progressive patients**. Waterfall plot showing the prediction (scores per patient) of PFS based on basal kinase profiles VSN normalized and compared to the actual patient progression classified for PFS at 9 months post-treatment initiation (**A**). Green: responder-correct, Blue: responder-fail, grey: non-responder-correct, Red: non-responder-fail. (**B**).

**Fig. 5:**
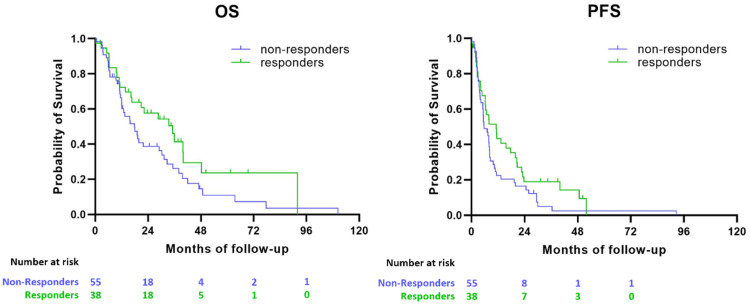
Kaplan–Meier plots showing OS (A) and PFS (B) of mRCC patients based on the PLSDA prediction score at 9 months (*p* = 0.079 and *p* = 0.086 for OS and PFS respectively).

To predict PFS of patients at 9 months post-initiation of treatment, supervised analysis using partial least squares discriminant analysis (PLS-DA) of basal kinome profiles was performed (Fig. 4). 55/93 (59 %) patients for whom follow-up was available 9 months post-initiation of treatment showed progression of disease, whereas 38/93 (41 %) patients did not show disease progression. When the basal kinase profiles were used as input to build a classification model (response prediction), only 67 % of non-responder patients and 53 % of responder patients were correctly classified (NPV of 0.673 (37/55), PPV of 0.526 (20/38), i.e., sensitivity 53 % (20/38), specificity 67 % (37/55), Fig. 4A, B). However, the misclassification rate (MCR) of 0.39 was lower than the permuted MCR (pMCR at *p* = 0.05), of 0.43 after 50 permutations. The PLSDA prediction score was used in Kaplan–Meier plots to show the correlation with PFS and OS (Fig. 5).

Sensitivity, specificity, positive predictive value (PPV) and negative predictive value (NPV) of patient samples (*n* = 93).

### Kinase Inhibition analysis

Because the specificity of the classifier was insufficient (61 % of patients correctly classified patients at 9-month PFS), we evaluated whether the *ex vivo* kinase inhibitory effect of sunitinib might result in a superior classifier, by spiking in sunitinib in the assay. Unsupervised hierarchical clustering of 109 peptide phosphorylation inhibition profiles of 99 mRCC patient samples revealed 2 clusters (Suppl Fig. S2). Group specific network mapping by Metacore analysis showed that the main difference between the clusters was immune response related (results not shown).

The patients with the shortest PFS and shortest OS showed the highest degree of *ex vivo* inhibition. However, the inhibition profiles poorly predicted 9-month PFS with only 57 % of patients correctly classified. Prediction of 12-month PFS was even poorer with only 55 % of patients correctly classified.

When patients with short OS (<12 months, *N* = 35) and patients with long OS (>24 months, *N* = 36) were selected, prediction was poor albeit that 2 peptides were significantly differentially phosphorylated: Myelin basic protein (MBP) and Fibroblast growth factor Receptor 3 (FGFR3) (*p* < 0.05, student T-test).

### Kinase activity of ccRCC and normal kidney tissue and sunitinib-related toxicity

Because intrinsic individual differences in PK activity may be related to TKI toxicity, we compared the kinase activity, both tyrosine kinase and serine‑threonine kinase (STK) of the patient samples from which tumor and adjacent corresponding normal kidney tissue was available (14 patients, Suppl figure S3).

Grade 3 sunitinib-related toxicity, defined as Common Terminology Criteria for Adverse Events [CTCAE version 4.0] > 3, was experienced by 7 patients, whereas 7 patients did not experience toxicity. Whereas PTK levels did not correlate with toxicity, STK levels did correlate with toxicity: STK levels were similar in ccRCC and normal kidney samples from patients that experienced grade 3 toxicity whereas in the population without toxicity STK levels were higher in ccRCC than in normal kidney (38 peptides significantly different phosphorylated, 1 peptide lower, 37 peptides more phosphorylated in ccRCC, Table 1).

**Table 1:** Phosphosite Analysis to correlate toxicity to tumor versus normal tissue kinase activity.

Assay type	STK	
Comparisons	Up	Down
No toxicity	37	1
Toxicity	3	1

Because each kinase phosphorylates multiple phosphosites on the PamChip we performed an Upstream Kinase Analysis (UKA) to address which kinases are responsible for the phosphorylation differences between tumor and normal tissue (Figure S4, Table S4). CHK2, CDKL1, PKA[alpha], Akt1/PKB[alpha], mTOR/FRAP, Akt2/PKB[beta], ATR, PKG1, RSK2 were the top 10 upstream kinases with higher activity in tumor than in normal kidney.

## Discussion

Multiple therapies for patients with mRCC are available. The current guidelines of the European Association of Urology prescribe sunitinib as one of the first-line treatment modalities for patients with metastasized ccRCC for patients not qualifying for IC treatment [5]. Thus far, stratification of patients is based on clinical parameters, and comparison of several risk models showed a concordance level of 66 %, indicating that a ceiling has been reached for clinical risk models to predict prognosis based solely on clinical factors. This includes their use in the era of targeted therapy [[17], [18], [19]]. In view of the variable response and the sequential use of therapies, it is becoming increasingly important to stratify patients to predict whether they will benefit from the chosen treatment modality, and predictive biomarkers are urgently needed. Extensive molecular profiling of ccRCC has revealed key molecular drivers and unique and diverse alterations [[20], [21], [22]]. Based on molecular profiling prognostic signatures have been proposed (e.g., [23], but thus far correlations with treatment outcome have been sparce [24]. Here we used kinomic profiling which measures the functional activity of the tyrosine and serine‑threonine kinases on peptide substrates. It is possible that these enzyme activities are more relevant than kinase transcript expression levels. We studied kinase levels of primary ccRCC tumors and showed that supervised clustering of basal kinome activity could correctly classify the 9 months PFS of mRCC patients with an accuracy of 61 %. The distinction of patients experiencing long sunitinib response versus patients experiencing shorter PFS is of importance as it can guide treatment choice, preventing treatment of poorly responding patient. Nevertheless, the level of accuracy of the classifier may not be sufficient to achieve implementation in clinical practice.

Unsupervised hierarchical clustering of kinomic profiles of primary ccRCC from mRCC patients revealed 3 major clusters related to cell adhesion, inflammation, and immune response. Patients with a cluster C profile performed better than cluster A patients. Pathway analysis of cluster C showed enrichment for VEGFR signaling, one of the sunitinib targets, which may explain the better treatment outcome. Patients with a cluster A profile performed poorly compared to clusters B and C. In cluster A immune signally pathways were enriched, and it is possible that these patients could benefit from checkpoint inhibitor treatment, but this needs to be studied carefully.

The 3 clusters described here differ slightly from the 3 clusters that were identified in kinome profiling of ccRCC patients with localized disease [14]: similar sized clusters related to translation initiation, immune response/cell adhesion and inflammation were distinguished. In our study the cluster related to cell adhesion was quite small (17/101, 17 %) and this may reflect the fact that all patients included had metastatic disease. Thus, events related to metastatic potential, intimately related to cell adhesion events, may be less apparent. In contrast to Andersons’ study [14], our largest cluster was related to inflammation (47/101, 47 %). Whether this difference reflects sample size or a reflection of the higher disease stage of our patient cohort is unknown. Selection for a more aggressive phenotype, including immune suppression, is more homogeneous in our population, and it is possible that this correlates with slightly different kinase profiles explaining the observed differences.

Basal kinase activity levels of patients with short PFS were substantially higher compared to patients who experienced extended PFS. When the peptide phosphorylation inhibition was evaluated *in vitro* in sunitinib spike-in experiments, patients with the shortest PFS showed the highest relative *ex vivo* inhibition. These patients also showed the highest kinase activity which may explain the higher relative *in vitro* inhibition. Nevertheless, despite this higher *ex vivo* inhibition, these patients performed poorly. This suggests that under these high kinase conditions the kinase activity of ccRCC is insufficiently inhibited by sunitinib to be beneficial. Interestingly, NOE et al. described that, based on day 21/day 0 lymphocyte ratio, patients with poor prognosis showed higher inhibitory effects [14]. This is in line with our finding showing that patients expressing high kinase levels perform poorly and that kinase levels may be used as predictive biomarker.

Unsupervised hierarchical clustering of the inhibition profiles revealed 2 clusters; the main difference between the clusters was immune response related. We next used the inhibition profiles to determine whether these clusters correlated with PFS and disappointingly no correlation with PFS was found.

STK analysis of paired normal kidney tissue and ccRCC revealed a substantial number of peptides that were significantly differentially phosphorylated. None of the top 10 upstream kinases overlapped with kinases described earlier by Anderson et al. who described differential phosphorylation of 20 peptides [14]. They predicted upregulation of multiple upstream MAPK and reduced CDK1 and RSK1-4 kinase activity with altered mTOR components in ccRCC. In contrast, we found upregulation of RSKT1,2 and mTOR in ccRCC. Tahiri et al. described kinase substrate differences between normal kidney and tumor with high activity of Src family kinases and the phosphoinositide-3-kinase (PI3K) pathway [25]. Neither in our study nor in the study of Anderson was this difference prominent. Tahiri included 9/23 tumors with sarcomatoid features, a patient population dissimilar to our population, which may explain the difference. Additionally, the difference may be explained by the small number of patients included in all studies and/or by tumor heterogeneity which is known to play a prominent role in ccRCC [26].

For patients experiencing grade 3 toxicity STK profiling showed similar STK levels in normal kidney tissue and the corresponding ccRCC. Hence, it appears that when kinase activity in tumor and normal tissue are similar, toxicity is more likely to occur and STK assays may help to predict sunitinib toxicity preventing unwanted toxicity necessitating drug cessation. However, PFS and OS of patients experiencing common adverse events is higher compared to patients experiencing less toxicity [27] and cumulative toxicity has been associated with a significantly greater median OS and PFS compared with those who experience one or no adverse events [28]. Therefore, although toxicity may be predictable by STK assays, a balanced decision is needed to judge whether toxicity is acceptable in view of the better treatment outcome. This could lead to a change in clinical management.

One limitation of our study is that we evaluated only one ccRCC sample per patient and sampling of multiple ccRCC regions may be needed to adequately judge the kinase profile of a particular ccRCC. It is well established that ccRCC is quite heterogeneous at the molecular level, with multiple clones present in primary ccRCC [26,29]. To address whether heterogeneity played a role in the kinase analysis, multiple samples were evaluated from a limited number of ccRCC. Kinase patterns were quite stable (results not shown), suggesting that despite genetic heterogeneity kinase activity is quite homogeneous within a single ccRCC. Nevertheless, whether more sampling can improve the level of accuracy of the classifier remains to be determined.

A second limitation is the fact that we performed the kinase assays on primary ccRCC which may not reflect the kinase activity of corresponding metastatic lesions. Clearly, prediction of the effects of TKI treatment of metastatic disease is required. It is possible that determination of kinase activities of ccRCC metastases may be more informative. However, metastasectomies for ccRCC are uncommon whereas primary ccRCC is generally readily available. In view of our results, kinase analysis of primary ccRCC was quite informative for stratification purposes, and moreover, if patients recur, kinase analysis of primary ccRCC may allow treatment stratification.

In 2011 by Poste discussed logistical and regulatory challenges in biomarker research [30]. Specifically, lack of standardization, insufficient sampling, sample size, heterogeneity in study cohort and lack of validation were mentioned as major hurdles. Here we attempted to minimize sampling error, include a homogeneous treatment group, and increase sample size through inclusion by dedicated EuroTARGET partners, in principle ensuring that samples were collected, annotated, stored, and analyzed under standardized conditions and accompanied by clinical information. Nevertheless, although all partners were heavily committed, the total number of patients that could be included was limited, also because during the study treatment management of mRCC shifted from TKI to alternative treatment modalities, hampering rapid inclusion. Moreover, fresh frozen material is required for the kinase assay, and this is logistically challenging, and some committed partners could not deliver material of sufficient quality. This again demonstrates the challenges facing biomarker research, apart from subsequent challenges faced when bringing a biomarker to clinical practice in conjunction with reimbursement. Nevertheless, improvement of treatment and reduction of health-care costs through deployment of biomarker is still potentially greater than in any other area of current medical research [30].

In conclusion, basal kinome activity of primary ccRCC can classify mRCC patients treated with sunitinib in long and short progression-free survivors with an accuracy of 61 % (9-months PFS), likely too low to include as prognostic or predictive biomarker. Then again, STK assays may help to predict sunitinib toxicity and guide clinical management. Three clusters of ccRCC were distinguished based on kinase levels which may reflect their aggressiveness. Additionally, it is conceivable that mRCC patients with a ccRCC tumor with an immune kinase signature are better candidates for checkpoint inhibitor or TKI/checkpoint inhibitor combination treatment. Intriguingly, combinations of TKI with checkpoint inhibitors have recently been included as standard of care in first and second line treatment of mRCC [4] and patient stratification based on kinase studies might improve treatment outcome, but this needs to be studied. Lastly, it might be more informative to pattern the kinase activity of ccRCC metastases since sunitinib effects on metastases determines PFS and OS.

## CRediT authorship contribution statement

**Jeannette C. Oosterwijk-Wakka:** Writing – review & editing, Writing – original draft, Visualization, Supervision, Methodology, Investigation, Conceptualization. **Liesbeth Houkes:** Writing – review & editing, Writing – original draft, Visualization, Validation, Methodology, Investigation, Formal analysis, Data curation, Conceptualization. **Loes F.M. van der Zanden:** Writing – review & editing, Data curation. **Lambertus A.L.M. Kiemeney:** Writing – review & editing, Resources, Funding acquisition, Conceptualization. **Kerstin Junker:** Writing – review & editing, Resources, Funding acquisition. **Anne Y Warren:** Writing – review & editing, Data curation. **Tim Eisen:** Writing – review & editing, Funding acquisition. **Ulrich Jaehde:** Writing – review & editing, Funding acquisition. **Marius T Radu:** Writing – review & editing, Funding acquisition. **Rob Ruijtenbeek:** Writing – review & editing, Methodology, Investigation, Funding acquisition, Conceptualization. **Egbert Oosterwijk:** Writing – review & editing, Writing – original draft, Supervision, Methodology, Investigation, Funding acquisition, Conceptualization.

## Declaration of competing interest

The authors declare the following financial interests/personal relationships which may be considered as potential competing interests:

L. Houkes and R Ruijtenbeek are employed by PamGene International B.V., 5211 HH 's-Hertogenbosch, The Netherlands.

The other authors declare that they have no competing interests.

## Appendix Supplementary materials

Image, application 1
