# Supplementary material for: Kinomic profiling to predict sunitinib response of patients with metastasized clear cell Renal Cell Carcinoma
Source: Neoplasia. 2024 Dec 25;60:101108. doi: 10.1016/j.neo.2024.101108 (PMC11732189; doi:10.1016/j.neo.2024.101108)
Supplement: Supplementary file 1 [file mmc1.docx]

Supplementary figures and tables

**Figure S1**. Z-score normalized per peptide

C

A

B


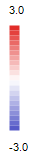

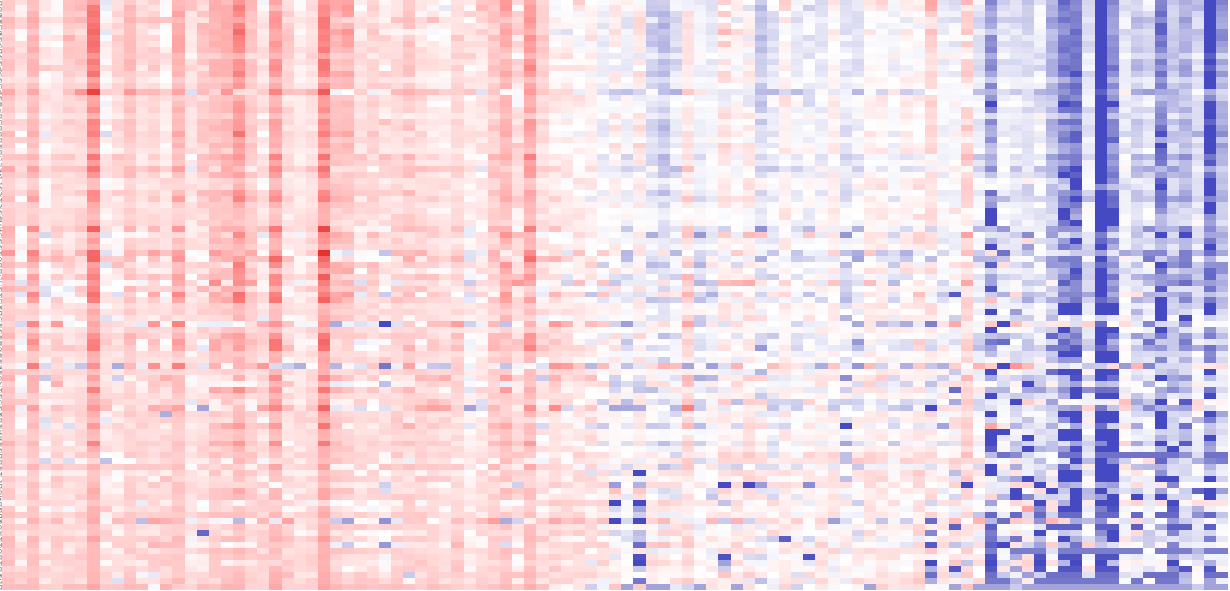


Figure S2


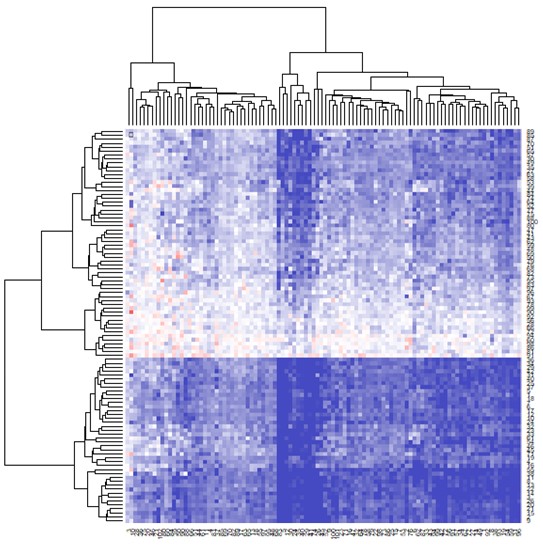

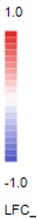


Unsupervised clustering of inhibition profiles of ccRCC samples from 99 patients. Individual patients are shown at the X-axis, Y-axis depicts inhibition of peptide phosphorylation at 0.3 μM sunitinib compared to uninhibited basal kinase activity.

Supplementary Figure S3


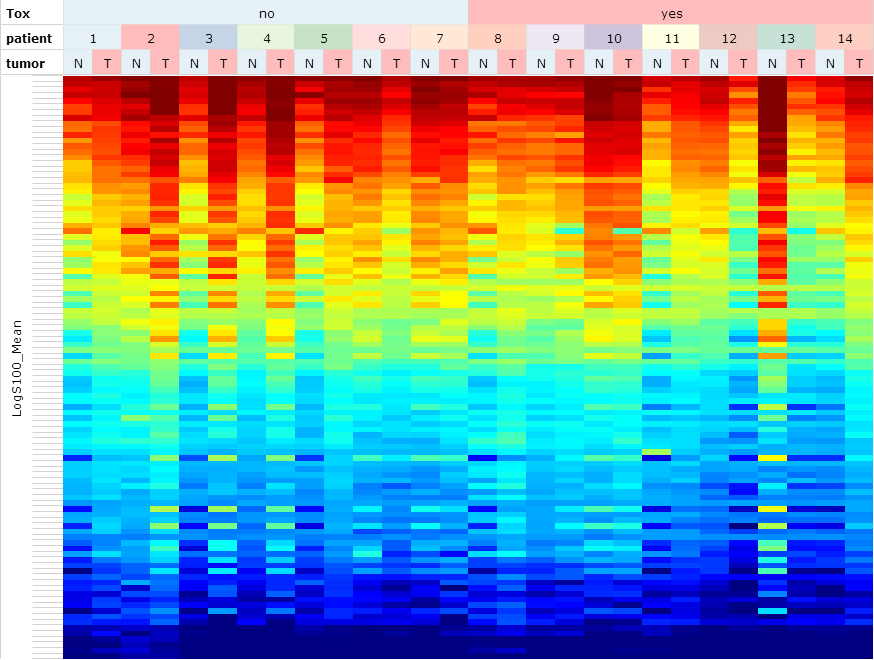

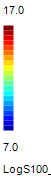


Figure S3. Serine-threonine kinase (STK)) activities from tumor and adjacent corresponding normal kidney tissue samples. Grade 3 sunitinib-related toxicity was experienced by 7 patients (yes), whereas 7 patients did not experience toxicity (no)

Supplementary Figure S4


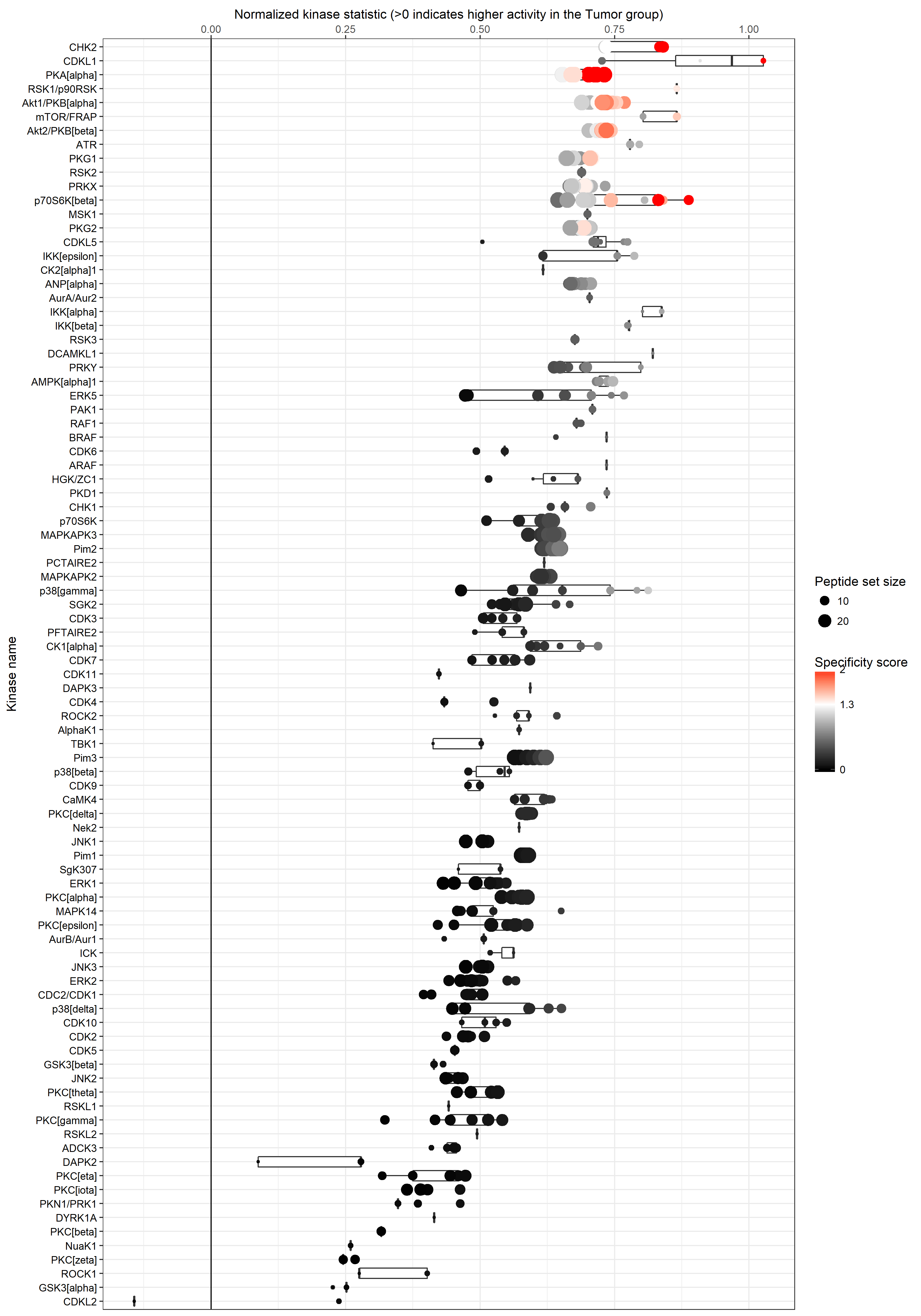


**Figure S4.** Upstream kinase analysis identifies kinases that might be responsible for the differences in phosphorylation profiles between normal kidney and ccRCC. Kinases at the top are most likely to be involved.

**Supplementary table S1A**

|  | **UniprotAccession** | **ID** | **p-value** | **delta** |  |
| --- | --- | --- | --- | --- | --- |
| 1 | P20963 | CD3Z_116_128 | 5,5E-10 | -0,51 | T-cell surface glycoprotein CD3 zeta chain |
| 2 | P19235 | EPOR_361_373 | 6,0E-10 | -0,38 | Erythropoietin receptor |
| 3 | P24941 | CDK2_8_20 | 2,6E-09 | -0,35 | Cyclin-dependent kinase 2 |
| 4 | P35222 | CTNB1_79_91 | 3,5E-09 | -0,51 | Catenin beta-1 |
| 5 | P09619 | PGFRB_572_584 | 7,9E-09 | -0,62 | Platelet-derived growth factor receptor beta |
| 6 | P16284 | PECA1_706_718 | 9,4E-09 | -0,30 | Platelet endothelial cell adhesion molecule |
| 7 | P16591 | FER_707_719 | 2,5E-08 | -0,28 | Tyrosine-protein kinase Fer |
| 8 | P46108 | CRK_214_226 | 3,0E-08 | -0,51 | Adapter molecule crk |
| 9 | P07332 | FES_706_718 | 2,1E-07 | -0,31 | Tyrosine-protein kinase Fes/Fps |
| 10 | P43403 | ZAP70_485_497 | 2,5E-07 | -0,25 | Tyrosine-protein kinase ZAP-70 |
| 11 | O60674 | JAK2_563_577 | 3,8E-07 | -0,22 | Tyrosine-protein kinase JAK2 |
| 12 | P20963 | CD3Z_146_158 | 4,0E-07 | -0,44 | T-cell surface glycoprotein CD3 zeta chain |
| 13 | P04049 | RAF1_332_344 | 5,3E-06 | -0,71 | RAF proto-oncogene serine/threonine-  protein kinase |
| 14 | P42685 | FRK_380_392 | 2,0E-05 | -0,21 | Tyrosine-protein kinase FRK |
| 15 | P35916 | VGFR3_1061_1073 | 3,0E-05 | -0,47 | Vascular endothelial growth factor  receptor 3 |
| 16 | P49023 | PAXI_111_123 | 3,3E-05 | -0,29 | Paxillin |
| 17 | P35968 | VGFR2_1168_1180 | 4,1E-05 | -0,39 | Vascular endothelial growth factor  receptor 2 |
| 18 | Q16620 | NTRK2_696_708 | 9,7E-05 | -0,40 | BDNF/NT-3 growth factors receptor |
| 19 | P06239 | LCK_387_399 | 1,2E-04 | -0,21 | Tyrosine-protein kinase Lck |
| 20 | P09619 | PGFRB_709_721 | 1,5E-04 | -0,16 | Platelet-derived growth factor receptor  beta |
| 21 | P17948 | VGFR1_1326_1338 | 2,1E-04 | -0,20 | Vascular endothelial growth factor  receptor 1 |
| 22 | Q04912 | RON_1346_1358 | 2,6E-04 | -0,29 | Macrophage-stimulating protein receptor |
| 23 | P09619 | PGFRB_1002_1014 | 3,0E-04 | -0,35 | Platelet-derived growth factor receptor  beta |
| 24 | P09619 | PGFRB_771_783 | 4,4E-04 | -0,16 | Platelet-derived growth factor receptor  beta |
| 25 | Q01406 | SRC8_CHICK_476_488 | 5,4E-04 | -0,25 | Src substrate protein p85 |
| 26 | Q14765 | STAT4_714_726 | 6,0E-04 | -0,25 | Signal transducer and activator of  transcription 4 |
| 27 | P23458 | JAK1_1015_1027 | 6,6E-04 | -0,16 | Tyrosine-protein kinase JAK1 |
| 28 | P20936 | RASA1_453_465 | 9,0E-04 | -0,17 | Ras GTPase-activating protein 1 |
| 29 | Q15375 | EPHA7_607_619 | 1,5E-03 | -0,19 | Ephrin type-A receptor 7 |
| 30 | P09619 | PGFRB_768_780 | 1,6E-03 | -0,21 | Platelet-derived growth factor receptor  beta |
| 31 | P07355 | ANXA2_17_29 | 1,6E-03 | -0,26 | Annexin A2 |
| 32 | P06401 | PRGR_786_798 | 1,7E-03 | -0,19 | Progesterone receptor |
| 33 | P19174 | PLCG1_764_776 | 3,0E-03 | -0,21 | 1-phosphatidylinositol 4,5-bisphosphate  phosphodiesterase gamma-1 |
| 34 | Q04912 | RON_1353_1365 | 3,0E-03 | -0,17 | Macrophage-stimulating protein receptor |
| 35 | O15530 | PDPK1_2_14 | 4,2E-03 | -0,26 | Synapsin II |
| 36 | P49023 | PAXI_24_36 | 6,2E-03 | -0,15 | Paxillin |
| 37 | P00533 | EGFR_1190_1202 | 6,6E-03 | -0,17 | Epidermal growth factor receptor |
| 38 | P07949 | RET_1022_1034 | 2,5E-02 | -0,21 | Proto-oncogene tyrosine-protein kinase  receptor Ret |

Peptides with significantly more tyrosine phosphorylation in cluster 1A compared to cluster 1B (vsn normalised). Delta indicates the fold change difference between the groups on VSN normalized data.

**Supplementary table S1B**

|  | **UniprotAccession** | **ID** | **p-value** | **delta** |  |
| --- | --- | --- | --- | --- | --- |
| 1 | Q06418 | TYRO3_679_691 | 4,6E-11 | 0,54 | Tyrosine-protein kinase receptor TYRO3 |
| 2 | Q15303 | ERBB4_1277_1289 | 7,2E-11 | 0,65 | Receptor tyrosine-protein kinase erbB-4 |
| 3 | Q07075 | AMPE_5_17 | 6,6E-08 | 0,46 | Glutamyl aminopeptidase |
| 4 | P35968 | VGFR2_944_956 | 1,3E-07 | 0,56 | Vascular endothelial growth factor receptor 2 |
| 5 | P02686 | MBP_263_275 | 1,4E-07 | 0,40 | Myelin basic protein |
| 6 | Q05516 | ZBT16_621_633 | 4,4E-07 | 0,30 | Zinc finger and BTB domain-containing protein 16 |
| 7 | P18206 | VINC_815_827 | 2,3E-06 | 0,35 | Vinculin |
| 8 | P21802 | FGFR2_762_774 | 2,6E-06 | 0,37 | Fibroblast growth factor receptor 2 |
| 9 | P00533 | EGFR_1165_1177 | 6,6E-06 | 0,35 | Epidermal growth factor receptor |
| 10 | Q06495 | NPT2A_501_513 | 9,8E-06 | 0,59 | Sodium-dependent phosphate transport protein 2A |
| 11 | P28482 | MK01_180_192 | 3,0E-05 | 0,40 | Mitogen-activated protein kinase 1 |
| 12 | Q06124 | PTN11_539_551 | 1,2E-04 | 0,40 | Tyrosine-protein phosphatase non-receptor type 11 |
| 13 | P62714 | PP2AB_297_309 | 1,6E-04 | 0,32 | Serine/threonine-protein phosphatase 2A catalytic subunit beta isoform |
| 14 | P53779 | MK10_216_228 | 2,1E-04 | 0,31 | Mitogen-activated protein kinase 10 |
| 15 | P06400 | RB_804_816 | 5,1E-04 | 0,23 | Retinoblastoma-associated protein |
| 16 | P53778 | MK12_178_190 | 9,2E-04 | 0,19 | Mitogen-activated protein kinase 12 |
| 17 | P02686 | MBP_259_271 | 1,5E-03 | 0,20 | Myelin basic protein |
| 18 | P42680 | TEC_512_524 | 1,7E-03 | 0,11 | Tyrosine-protein kinase Tec |
| 19 | P02686 | MBP_198_210 | 7,5E-03 | 0,22 | Myelin basic protein |
| 20 | P04626 | ERBB2_1241_1253 | 7,8E-03 | 0,16 | Receptor tyrosine-protein kinase erbB-2 |
| 21 | P54762 | EPHB1_921_933 | 1,1E-02 | 0,33 | Ephrin type-B receptor 1 |
| 22 | P22607 | FGFR3_753_765 | 2,0E-02 | 0,21 | Fibroblast growth factor receptor 3 |
| 23 | P35968 | VGFR2_1046_1058 | 2,4E-02 | 0,15 | Vascular endothelial growth factor receptor 2 |
| 24 | P50613 | CDK7_157_169 | 3,3E-02 | 0,27 | Cyclin-dependent kinase 7 |

Peptides with significantly more tyrosine phosphorylation in cluster 1B compared to cluster 1A (vsn normalised). Delta indicates the fold change difference between the groups on VSN normalized data.

**Supplementary table S1C**

|  | **UniprotAccession** | **ID** | **p-value** | **delta** |  |
| --- | --- | --- | --- | --- | --- |
| 1 | P22681 | CBL_693_705 | 1,7E-07 | -1,55 | E3 ubiquitin-protein ligase CBL |
| 2 | P12694 | ODBA_340_352 | 5,8E-06 | -0,71 | 2-oxoisovalerate dehydrogenase subunit alpha, mitochondrial |
| 3 | P09619 | PGFRB_1014_1028 | 2,6E-05 | -0,63 | Platelet-derived growth factor receptor beta |
| 4 | P04083 | ANXA1_14_26 | 1,2E-05 | -0,58 | Annexin A1 |
| 5 | P09619 | PGFRB_709_721 | 8,1E-06 | -0,49 | Platelet-derived growth factor receptor beta |
| 6 | O43561 | LAT_194_206 | 5,3E-04 | -0,49 | Linker for activation of T-cells family member 1 |
| 7 | P20963 | CD3Z_116_128 | 4,2E-04 | -0,48 | T-cell surface glycoprotein CD3 zeta chain |
| 8 | P29317 | EPHA2_765_777 | 2,4E-04 | -0,48 | Ephrin type-A receptor 2 |
| 9 | P09619 | PGFRB_1002_1014 | 7,9E-03 | -0,45 | Platelet-derived growth factor receptor beta |
| 10 | P54762 | EPHB1_771_783 | 1,3E-03 | -0,44 | Ephrin type-B receptor 1 |
| 11 | P11912 | CD79A_181_193 | 6,1E-03 | -0,39 | B-cell antigen receptor complex-associated protein alpha chain |
| 12 | O43561 | LAT_249_261 | 5,6E-04 | -0,39 | Linker for activation of T-cells family member 1 |
| 13 | P20963 | CD3Z_146_158 | 5,1E-03 | -0,37 | T-cell surface glycoprotein CD3 zeta chain |
| 14 | P46108 | CRK_214_226 | 3,2E-02 | -0,34 | Adapter molecule crk |
| 15 | P27986 | P85A_600_612 | 1,1E-03 | -0,34 | Phosphatidylinositol 3-kinase regulatory subunit alpha |
| 16 | P43405 | KSYK_518_530 | 1,0E-04 | -0,32 | Tyrosine-protein kinase SYK |
| 17 | Q01406 | SRC8_CHICK_492_504 | 8,9E-03 | -0,30 | Src substrate protein p85 |
| 18 | P07355 | ANXA2_17_29 | 1,4E-02 | -0,29 | Annexin A2 |
| 19 | P35968 | VGFR2_989_1001 | 5,4E-03 | -0,28 | Vascular endothelial growth factor  receptor 2 |
| 20 | P19235 | EPOR_419_431 | 1,6E-02 | -0,27 | Erythropoietin receptor |
| 21 | P21709 | EPHA1_774_786 | 5,1E-03 | -0,26 | Ephrin type-A receptor 1 |
| 22 | P08581 | MET_1227_1239 | 1,8E-03 | -0,24 | Hepatocyte growth factor receptor |
| 23 | P09619 | PGFRB_771_783 | 1,2E-02 | -0,23 | Platelet-derived growth factor receptor beta |
| 24 | P16284 | PECA1_706_718 | 7,7E-03 | -0,22 | Platelet endothelial cell adhesion molecule |
| 25 | P19235 | EPOR_361_373 | 4,4E-02 | -0,21 | Erythropoietin receptor |
| 26 | P23458 | JAK1_1015_1027 | 5,8E-03 | -0,20 | Tyrosine-protein kinase JAK1 |
| 27 | P49023 | PAXI_24_36 | 1,8E-02 | -0,20 | Paxillin |
| 28 | P05787 | K2C8_425_437 | 2,3E-02 | -0,19 | Keratin, type II cytoskeletal 8 |
| 29 | P11171 | 41_654_666 | 5,5E-03 | -0,17 | Protein 4.1 |

Peptides with significantly more tyrosine phosphorylation in cluster 1B compared to cluster 1C (vsn normalised). Delta indicates the fold change difference between the groups on VSN normalized data.

**Supplementary table S1D**

|  | **UniprotAccession** | **ID** | **p-value** | **delta** |  |
| --- | --- | --- | --- | --- | --- |
| 1 | P42680 | TEC_512_524 | 1,2E-02 | 0,14 | Tyrosine-protein kinase Tec |
| 2 | P54764 | EPHA4_589_601 | 4,5E-02 | 0,17 | Ephrin type-A receptor 4 |
| 3 | Q13164 | MK07_211_223 | 3,3E-02 | 0,23 | Mitogen-activated protein kinase 7 |
| 4 | P17948 | VGFR1_1040_1052 | 4,4E-03 | 0,28 | Vascular endothelial growth factor receptor 1 |
| 5 | P54760 | EPHB4_583_595 | 4,0E-04 | 0,30 | Ephrin type-A receptor 4 |
| 6 | P02686 | MBP_259_271 | 1,5E-02 | 0,30 | Myelin basic protein |
| 7 | O43602 | DCX_109_121 | 9,3E-03 | 0,32 | Notch homolog 2 N-terminal-like protein A |
| 8 | Q05516 | ZBT16_621_633 | 5,1E-03 | 0,37 | Zinc finger and BTB domain-containing protein 16 |
| 9 | Q07075 | AMPE_5_17 | 5,7E-03 | 0,38 | Glutamyl aminopeptidase |
| 10 | P35968 | VGFR2_1046_1058 | 1,8E-03 | 0,39 | Vascular endothelial growth factor receptor 2 |
| 11 | Q06495 | NPT2A_501_513 | 3,8E-02 | 0,46 | Sodium-dependent phosphate transport protein 2A |
| 12 | P35968 | VGFR2_944_956 | 1,9E-03 | 0,47 | Vascular endothelial growth factor receptor 2 |
| 13 | P11362 | FGFR1_761_773 | 3,8E-02 | 0,47 | Fibroblast growth factor receptor 1 |
| 14 | P62714 | PP2AB_297_309 | 2,1E-04 | 0,48 | Serine/threonine-protein phosphatase 2A catalytic  subunit beta isoform |
| 15 | P54762 | EPHB1_921_933 | 4,4E-02 | 0,48 | Ephrin type-B receptor 1 |

Peptides with significantly more tyrosine phosphorylation in cluster 1C compared to cluster 1B (vsn normalised). Delta indicates the fold change difference between the groups on VSN normalized data.

**Supplementary table S2A**

|  | **Uniprot ID** | **Kinase** | **Mean specificity Score** | **Mean significance score** | **Median final score** | **Median kinase statistic** | **# Peptides** | **Q-score** |
| --- | --- | --- | --- | --- | --- | --- | --- | --- |
| 1 | Q16288 | TRKC | 1,91 | 2,7 | 4,7 | -0,54 | 11 | 2,0E-05 |
| 2 | P06241 | Fyn | 1,53 | 2,7 | 4,17 | -0,5 | 10 | 6,8E-05 |
| 3 | P43403 | ZAP70 | 1,17 | 2,7 | 3,94 | -0,3 | 37 | 1,1E-04 |
| 4 | P12931 | Src | 1,36 | 2,7 | 3,92 | -0,36 | 25 | 1,2E-04 |
| 5 | P04629 | TRKA | 1,1 | 2,7 | 3,85 | -0,45 | 8 | 1,4E-04 |
| 6 | P16591 | Fer | 0,79 | 1,61 | 3,65 | -0,51 | 7 | 2,2E-04 |
| 7 | P54764 | EphA4 | 0,71 | 2,7 | 3,57 | -0,56 | 3 | 2,7E-04 |
| 8 | Q04912 | Ron | 0,89 | 2,7 | 3,53 | -0,43 | 6 | 3,0E-04 |
| 9 | P08581 | Met | 0,65 | 2,7 | 3,36 | -0,27 | 18 | 4,4E-04 |
| 10 | P43405 | Syk | 0,85 | 2,7 | 3,36 | -0,24 | 44 | 4,4E-04 |
| 11 | P51451 | BLK | 0,71 | 2,7 | 3,34 | -0,29 | 15 | 4,6E-04 |
| 12 | Q16620 | TRKB | 0,87 | 2,7 | 3,31 | -0,29 | 12 | 4,9E-04 |
| 13 | P08631 | HCK | 0,68 | 2,7 | 3,3 | -0,25 | 18 | 5,0E-04 |
| 14 | P06239 | Lck | 0,56 | 2,7 | 3,3 | -0,27 | 18 | 5,0E-04 |
| 15 | P21709 | EphA1 | 0,56 | 2,7 | 3,27 | -0,43 | 4 | 5,4E-04 |
| 16 | P41240 | CSK | 0,48 | 2,53 | 3,25 | -0,24 | 28 | 5,6E-04 |
| 17 | P07947 | Yes | 0,67 | 2,7 | 3,2 | -0,25 | 16 | 6,3E-04 |
| 18 | P30530 | Axl | 0,46 | 2,7 | 3,17 | -0,22 | 35 | 6,8E-04 |
| 19 | P06213 | InSR | 0,47 | 2,7 | 3,16 | -0,24 | 14 | 6,9E-04 |
| 20 | P22455 | FGFR4 | 0,51 | 2,39 | 3,15 | -0,28 | 11 | 7,1E-04 |
| 21 | Q9UM73 | ALK | 0,46 | 2,7 | 3,15 | -0,23 | 26 | 7,1E-04 |
| 22 | P42680 | TEC | 0,45 | 2,7 | 3,13 | -0,23 | 24 | 7,4E-04 |
| 23 | P07948 | Lyn | 0,38 | 2,62 | 3,11 | -0,24 | 14 | 7,8E-04 |
| 24 | P42679 | CTK | 0,56 | 2,7 | 3,11 | -0,22 | 20 | 7,8E-04 |
| 25 | P29376 | LTK | 0,55 | 2,7 | 3,08 | -0,23 | 10 | 8,3E-04 |
| 26 | P08069 | IGF1R | 0,46 | 2,7 | 3,07 | -0,24 | 9 | 8,5E-04 |
| 27 | P51813 | Etk/BMX | 0,38 | 2,7 | 3,07 | -0,23 | 19 | 8,5E-04 |
| 28 | Q06418 | Tyro3/Sky | 0,36 | 2,7 | 3,04 | -0,2 | 28 | 9,1E-04 |
| 29 | P00519 | Abl | 0,28 | 2,7 | 3,01 | -0,2 | 33 | 9,8E-04 |
| 30 | Q05397 | FAK1 | 0,33 | 2,7 | 3 | -0,19 | 25 | 1,0E-03 |
| 31 | P42685 | FRK | 0,32 | 2,7 | 2,99 | -0,2 | 31 | 1,0E-03 |
| 32 | P16234 | PDGFR[alpha] | 0,29 | 2,7 | 2,99 | -0,22 | 5 | 1,0E-03 |
| 33 | P09619 | PDGFR[beta] | 0,31 | 2,7 | 2,98 | -0,19 | 11 | 1,0E-03 |
| 34 | Q14289 | FAK2 | 0,32 | 2,7 | 2,97 | -0,19 | 21 | 1,1E-03 |
| 35 | P42684 | Arg | 0,31 | 2,7 | 2,94 | -0,18 | 31 | 1,1E-03 |
| 36 | Q12866 | Mer | 0,34 | 2,7 | 2,93 | -0,17 | 31 | 1,2E-03 |
| 37 | Q13882 | Brk | 0,19 | 2,7 | 2,9 | -0,18 | 38 | 1,3E-03 |
| 38 | Q9H3Y6 | Srm | 0,09 | 2,7 | 2,78 | -0,13 | 29 | 1,7E-03 |
| 39 | P54756 | EphA5 | 0,28 | 2,4 | 2,68 | -0,23 | 3 | 2,1E-03 |
| 40 | P11362 | FGFR1 | 0,26 | 2,02 | 2,65 | -0,18 | 7 | 2,2E-03 |
| 41 | P42681 | TXK | 0,23 | 2,13 | 2,49 | -0,11 | 20 | 3,2E-03 |
| 42 | P07332 | Fes | 0,24 | 2,28 | 2,41 | -0,15 | 9 | 3,9E-03 |
| 43 | Q08881 | ITK | 0,11 | 1,92 | 2,4 | -0,13 | 19 | 4,0E-03 |
| 44 | P21802 | FGFR2 | 0,42 | 1,7 | 2,32 | -0,21 | 5 | 4,8E-03 |
| 45 | Q13308 | CCK4/PTK7 | 0,18 | 2,07 | 2,19 | -0,18 | 3 | 6,5E-03 |
| 46 | P04626 | HER2 | 0,14 | 1,64 | 1,8 | -0,08 | 19 | 1,6E-02 |
| 47 | P22607 | FGFR3 | 0,3 | 1,56 | 1,56 | -0,15 | 5 | 2,8E-02 |

Kinases that are more active in cluster 1A compared to cluster 1B.

The mean specificity score is a ranking score for which kinases are most likely involved based on the peptide set. The mean significance score is a ranking score for which kinases are most likely involved based on the profile differences between the sample groups. The median final score is a ranking score for the probability that the activity of a specific kinase is higher in cluster A The median kinase statistic indicates the difference in phosphorylation signal between cluster A compared to cluster B.

The number of peptides indicates the size of the set of target peptides of a particular kinase in the analysis. The q-score is a combined specificity and significance score that indicates the chance that the kinase signal is not specific for the set of peptides and samples. (q-score is 10E-(median final score) which is redundancy in this table)

**Supplementary table S2B**

|  | **Uniprot ID** | **Kinase** | **Mean specificity Score** | **Mean significance score** | **Median final score** | **Median kinase statistic** | **# Peptides** | **Q-score** |
| --- | --- | --- | --- | --- | --- | --- | --- | --- |
| 1 | P06241 | **Fyn** | 1,5 | 2,7 | 4,36 | -0,54 | 10 | 4,4E-05 |
| 2 | Q04912 | Ron | 0,8 | 2,67 | 3,53 | -0,46 | 6 | 3,0E-04 |
| 3 | P29320 | EphA3 | 0,56 | 2,7 | 3,26 | -0,41 | 3 | 5,5E-04 |
| 4 | P29317 | EphA2 | 0,4 | 1,99 | 2,61 | -0,39 | 4 | 2,5E-03 |
| 5 | P08631 | HCK | 1,18 | 2,35 | 4,02 | -0,38 | 18 | 9,5E-05 |
| 6 | P51451 | BLK | 0,82 | 2,7 | 3,47 | -0,36 | 15 | 3,4E-04 |
| 7 | P43405 | **Syk** | 1,57 | 2,7 | 4,32 | -0,32 | 44 | 4,8E-05 |
| 8 | P29376 | LTK | 0,81 | 2,7 | 3,3 | -0,32 | 10 | 5,0E-04 |
| 9 | Q16288 | TRKC | 0,52 | 1,99 | 2,4 | -0,32 | 11 | 4,0E-03 |
| 10 | P43403 | ZAP70 | 1,04 | 2,7 | 3,71 | -0,31 | 37 | 1,9E-04 |
| 11 | P07947 | Yes | 0,6 | 2,7 | 3,32 | -0,31 | 16 | 4,8E-04 |
| 12 | P21860 | HER3 | 0,53 | 2,7 | 3,24 | -0,31 | 22 | 5,8E-04 |
| 13 | P06239 | Lck | 0,7 | 2,7 | 3,34 | -0,3 | 18 | 4,6E-04 |
| 14 | P54756 | EphA5 | 0,35 | 2,4 | 2,75 | -0,3 | 3 | 1,8E-03 |
| 15 | P42685 | FRK | 0,64 | 2,7 | 3,36 | -0,29 | 31 | 4,4E-04 |
| 16 | P00519 | Abl | 0,49 | 2,7 | 3,24 | -0,29 | 33 | 5,8E-04 |
| 17 | P04629 | TRKA | 0,41 | 1,68 | 2,09 | -0,29 | 8 | 8,1E-03 |
| 18 | P06213 | InSR | 0,49 | 2,7 | 3,15 | -0,28 | 14 | 7,1E-04 |
| 19 | P09769 | Fgr | 0,44 | 2,53 | 3,14 | -0,28 | 6 | 7,2E-04 |
| 20 | Q13308 | CCK4/PTK7 | 0,3 | 2,6 | 3 | -0,28 | 3 | 1,0E-03 |
| 21 | P21709 | EphA1 | 0,3 | 1,2 | 1,64 | -0,28 | 4 | 2,3E-02 |
| 22 | P12931 | Src | 0,51 | 2,2 | 2,9 | -0,27 | 25 | 1,3E-03 |
| 23 | H0Y8A4 | RYK | 0,33 | 1,84 | 2,05 | -0,27 | 5 | 8,9E-03 |
| 24 | Q06418 | Tyro3/Sky | 0,44 | 2,7 | 3,11 | -0,26 | 28 | 7,8E-04 |
| 25 | Q9UM73 | ALK | 0,38 | 2,7 | 3,09 | -0,25 | 26 | 8,1E-04 |
| 26 | P42679 | CTK | 0,69 | 2,7 | 3,03 | -0,24 | 20 | 9,3E-04 |
| 27 | P14616 | IRR | 0,27 | 1,21 | 1,53 | -0,24 | 3 | 3,0E-02 |
| 28 | P08581 | Met | 0,27 | 2,13 | 2,27 | -0,23 | 18 | 5,4E-03 |
| 29 | Q14289 | FAK2 | 0,31 | 2,34 | 2,98 | -0,22 | 21 | 1,0E-03 |
| 30 | Q05397 | FAK1 | 0,22 | 2,65 | 2,89 | -0,22 | 25 | 1,3E-03 |
| 31 | P08069 | IGF1R | 0,29 | 1,8 | 1,99 | -0,22 | 9 | 1,0E-02 |
| 32 | P51813 | Etk/BMX | 0,2 | 2,11 | 2,85 | -0,2 | 19 | 1,4E-03 |
| 33 | P42680 | TEC | 0,16 | 2,6 | 2,84 | -0,2 | 24 | 1,4E-03 |
| 34 | Q06187 | BTK | 0,2 | 1,43 | 1,67 | -0,2 | 11 | 2,1E-02 |
| 35 | Q16620 | TRKB | 0,23 | 1,32 | 1,52 | -0,2 | 12 | 3,0E-02 |
| 36 | Q13882 | Brk | 0,11 | 2,7 | 2,8 | -0,19 | 38 | 1,6E-03 |
| 37 | Q12866 | Mer | 0,12 | 2,5 | 2,82 | -0,18 | 31 | 1,5E-03 |
| 38 | Q9H3Y6 | Srm | 0,09 | 2,53 | 2,75 | -0,18 | 29 | 1,8E-03 |
| 39 | P07948 | Lyn | 0,33 | 2,06 | 2,08 | -0,18 | 14 | 8,3E-03 |
| 40 | P16234 | PDGFR[alpha] | 0,2 | 1,41 | 1,6 | -0,18 | 5 | 2,5E-02 |
| 41 | P30530 | Axl | 0,08 | 2,38 | 2,56 | -0,17 | 35 | 2,8E-03 |
| 42 | P42681 | TXK | 0,16 | 2,31 | 2,5 | -0,16 | 20 | 3,2E-03 |
| 43 | P09619 | PDGFR[beta] | 0,31 | 1,98 | 1,61 | -0,16 | 11 | 2,5E-02 |
| 44 | P42684 | Arg | 0,11 | 2,09 | 2,42 | -0,15 | 31 | 3,8E-03 |
| 45 | P07332 | Fes | 0,21 | 1,36 | 1,22 | -0,15 | 9 | 6,0E-02 |
| 46 | P10721 | Kit | 0,16 | 1,26 | 1,11 | -0,12 | 8 | 7,8E-02 |
| 47 | P54764 | EphA4 | 0,11 | 0,54 | 0,61 | -0,12 | 3 | 2,5E-01 |
| 48 | Q08881 | ITK | 0,07 | 1,3 | 1,28 | -0,11 | 19 | 5,2E-02 |
| 49 | P07949 | Ret | 0,09 | 0,95 | 0,84 | -0,11 | 12 | 1,4E-01 |
| 50 | P16591 | Fer | 0,11 | 0,63 | 0,51 | -0,11 | 7 | 3,1E-01 |
| 51 | P41240 | CSK | 0,01 | 0,85 | 0,86 | -0,1 | 28 | 1,4E-01 |
| 52 | P11362 | FGFR1 | 0,2 | 1,23 | 1,29 | -0,09 | 7 | 5,1E-02 |
| 53 | P00533 | EGFR | 0,02 | 1,49 | 1 | -0,08 | 27 | 1,0E-01 |
| 54 | P23458 | JAK1~b | 0,08 | 0,28 | 0,36 | -0,08 | 3 | 4,4E-01 |
| 55 | P22455 | FGFR4 | 0,05 | 0,42 | 0,43 | -0,07 | 11 | 3,7E-01 |
| 56 | O60674 | JAK2 | 0,04 | 0,56 | 0,44 | -0,05 | 14 | 3,6E-01 |
| 57 | Q15303 | HER4 | 0,05 | 0,52 | 0,32 | -0,03 | 17 | 4,8E-01 |
| 58 | P04626 | HER2 | 0,02 | 0,25 | 0,2 | -0,02 | 19 | 6,3E-01 |

Kinases that are more active in cluster 1B compared to cluster 1C (vsn).

**Supplementary table S3A**

Pathway maps basal cluster A, vsn normalized

**Supplementary table S3B**

Pathway maps basal cluster B vs cluster A, vsn normalized

**Supplementary table S3C**

Pathway maps basal cluster B vs cluster C, vsn normalized

**Supplementary table S3D**

Pathway maps basal cluster C, vsn normalized

Supplementary table S4

| Kinase Uniprot ID | Kinase | Mean Specificity Score | Mean Significance Score | Median Final score | Median Kinase Statistic | # peptides | Q-score |
| --- | --- | --- | --- | --- | --- | --- | --- |
| O96017 | CHK2 | 1.96 | 1.65 | 3.85 | 0.84 | 16 | 1.4E-04 |
| Q00532 | CDKL1 | 1.56 | 1.79 | 3.35 | 0.97 | 4 | 4.5E-04 |
| P17612 | PKA[alpha] | 1.83 | 1.45 | 3.17 | 0.70 | 52 | 6.7E-04 |
| Q15349 | RSK1/p90RSK | 1.28 | 1.84 | 3.11 | 0.87 | 5 | 7.8E-04 |
| P31749 | Akt1/PKB[alpha | 1.39 | 1.45 | 2.87 | 0.73 | 26 | 1.4E-03 |
| P42345 | mTOR/FRAP | 1.20 | 1.51 | 2.85 | 0.87 | 6 | 1.4E-03 |
| P31751 | Akt2/PKB[beta] | 1.32 | 1.45 | 2.77 | 0.73 | 26 | 1.7E-03 |
| Q13535 | ATR | 0.91 | 1.78 | 2.70 | 0.78 | 7 | 2.0E-03 |
| Q13976 | PKG1 | 1.17 | 1.31 | 2.49 | 0.70 | 35 | 3.2E-03 |
| P51812 | RSK2 | 0.54 | 1.88 | 2.45 | 0.69 | 9 | 3.6E-03 |
| P51817 | PRKX | 1.01 | 1.37 | 2.43 | 0.69 | 31 | 3.7E-03 |
| Q9UBS0 | p70S6K[beta] | 1.46 | 1.38 | 2.35 | 0.74 | 23 | 4.4E-03 |
| O75582 | MSK1 | 0.54 | 1.78 | 2.34 | 0.70 | 7 | 4.5E-03 |
| Q13237 | PKG2 | 1.02 | 1.28 | 2.31 | 0.69 | 32 | 4.9E-03 |
| O76039 | CDKL5 | 0.59 | 1.56 | 2.30 | 0.72 | 6 | 5.0E-03 |
| Q14164 | IKK[epsilon] | 0.63 | 1.66 | 2.26 | 0.76 | 7 | 5.5E-03 |
| P68400 | CK2[alpha]1 | 0.27 | 1.96 | 2.23 | 0.62 | 3 | 5.9E-03 |
| P16066 | ANP[alpha] | 0.66 | 1.49 | 2.20 | 0.67 | 19 | 6.3E-03 |
| O14965 | AurA/Aur2 | 0.50 | 1.64 | 2.16 | 0.70 | 5 | 6.9E-03 |
| O15111 | IKK[alpha] | 0.82 | 1.32 | 2.16 | 0.84 | 4 | 6.9E-03 |
| O14920 | IKK[beta] | 0.65 | 1.51 | 2.15 | 0.78 | 4 | 7.1E-03 |
| Q15418 | RSK3 | 0.50 | 1.65 | 2.11 | 0.68 | 9 | 7.8E-03 |
| O15075 | DCAMKL1 | 0.72 | 1.38 | 2.09 | 0.82 | 3 | 8.1E-03 |
| O43930 | PRKY | 0.58 | 1.50 | 2.06 | 0.69 | 10 | 8.6E-03 |
| Q13131 | AMPK[alpha]1 | 0.82 | 1.30 | 2.05 | 0.74 | 10 | 9.0E-03 |
| Q13164 | ERK5 | 0.38 | 1.52 | 1.98 | 0.66 | 13 | 1.0E-02 |

Kinases that are more active in ccRCC than normal tissue in pts experiencing no toxicity to sunitinib
